# The Effect of Positive End-Expiratory Pressure on Lung Micromechanics Assessed by Synchrotron Radiation Computed Tomography in an Animal Model of ARDS

**DOI:** 10.3390/jcm8081117

**Published:** 2019-07-28

**Authors:** Gaetano Scaramuzzo, Ludovic Broche, Mariangela Pellegrini, Liisa Porra, Savino Derosa, Angela Principia Tannoia, Andrea Marzullo, João Batista Borges, Sam Bayat, Alberto Bravin, Anders Larsson, Gaetano Perchiazzi

**Affiliations:** 1 Department of Morphology, Surgery and Experimental Medicine, Ferrara University, 44121 Ferrara, Italy; 2 Hedenstierna Laboratory, Department of Surgical Sciences, Uppsala University, 75185 Uppsala, Sweden; 3 Department of Anesthesia and Intensive Care, Uppsala University Hospital, 75185 Uppsala, Sweden; 4 Department of Physics, University of Helsinki, FI-00014 Helsinki, Finland; 5 Helsinki University Hospital, FI-00029 Helsinki, Finland; 6 Department of Emergency and Organ Transplant, Bari University, 70124 Bari, Italy; 7 Centre for Human and Applied Physiological Sciences, Faculty of Sciences and Medicine, King’s College, London WC2R 2LS, UK; 8 The European Synchrotron Radiation Facility, 38043 Grenoble, France; 9 INSERM UA7, Synchrotron Radiation for Biomedicine (STROBE) Laboratory, University of Grenoble Alpes, 38043 Grenoble, France

**Keywords:** ARDS, recruitment, VILI, alveoli, kinetics, synchrotron radiation computed tomography

## Abstract

Modern ventilatory strategies are based on the assumption that lung terminal airspaces act as isotropic balloons that progressively accommodate gas. Phase contrast synchrotron radiation computed tomography (PCSRCT) has recently challenged this concept, showing that in healthy lungs, deflation mechanisms are based on the sequential de-recruitment of airspaces. Using PCSRCT scans in an animal model of acute respiratory distress syndrome (ARDS), this study examined whether the numerosity (ASnum) and dimension (ASdim) of lung airspaces change during a deflation maneuver at decreasing levels of positive end-expiratory pressure (PEEP) at 12, 9, 6, 3, and 0 cmH_2_O. Deflation was associated with significant reduction of ASdim both in the whole lung section (passing from from 13.1 ± 2.0 at PEEP 12 to 7.6 ± 4.2 voxels at PEEP 0) and in single concentric regions of interest (ROIs). However, the regression between applied PEEP and ASnum was significant in the whole slice (ranging from 188 ± 52 at PEEP 12 to 146.4 ± 96.7 at PEEP 0) but not in the single ROIs. This mechanism of deflation in which reduction of ASdim is predominant, differs from the one observed in healthy conditions, suggesting that the peculiar alveolar micromechanics of ARDS might play a role in the deflation process.

## 1. Introduction

Although the prognosis of patients suffering from acute respiratory distress syndrome (ARDS) has improved [1] with the introduction of protective mechanical ventilation in clinical practice [2], ventilator-induced lung injury (VILI) [3] can amplify local and systemic inflammatory responses and worsen the clinical course. During positive pressure mechanical ventilation, the gas mixture is moved through the airways [4] down to the alveoli. It is commonly believed that the macroscopic lung change in volume derives from the isotropic inflation of the single alveoli [5,6], where changes in alveolar dimensions derive from the uniform scaling of all dimensions [7] but how alveoli behave microscopically is still only partially understood. In this respect, the present researchers recently observed [8] that in healthy lungs, airspaces reacted to deflation by changing their number more than their dimension. Moreover, the outer subpleural airspaces acted differently from the more internal ones.

Researchers have already used computed tomography (CT) to demonstrate that, in healthy lungs [9], multiple asynchronous events of alveolar recruitment and inflation occur through the whole inspiratory portion of the pressure–volume curve [10]. Animal models have revealed how, during inspiration, groups of alveoli expand while others contract [10,11,12].

In ARDS, which heterogeneously affects the lung parenchyma, the co-existence of neighboring areas with different time constants creates ridges of strain gradients in close proximity [13]. 

In fact, alveoli that have different time constants inflate at different speeds and maintain different volumes during the respiratory cycle [14]. Computation of time constants is very useful for a thorough assessment of lung mechanics [15], however their estimation in the clinical context must take into consideration also the effects of tubing resistance [16] and the density of the respiratory gases [17].

Although lung stress and strain are the principal determinants of VILI, their magnitude cannot be inferred by the measurement of plateau pressure and tidal volume at airway opening [18]. Furthermore, regional inhomogeneity computed on CT scans by volume gradients [19] or quadtree decomposition of compliance maps [10] is thought to act as a stress raiser, i.e., a local multiplier of the effects of pressure [20], in the model of lung fiber networks proposed by Mead et al. [21].

Moreover, during the development of VILI, microscopic air distribution is thought to be more complex and can also be influenced by the ventro-dorsal gradient of pressures as a consequence of gravitational edema [22], the local arrangement of bronchial-vascular blocks [23], and the distance from the pleural surface [8]. The poor understanding of this phenomenon derives from the difficulty of imaging the entire lung at high resolution, in vivo, and with an intact chest wall–lung complex. These conditions cannot be fulfilled by conventional imaging methods like CT [24,25], He-hyperpolarized nuclear magnetic resonance [26], or in vivo subpleural microscopy [27,28]. Phase contrast synchrotron radiation computed tomography (PCSRCT) allows us to overcome these limits by using highly coherent monochromatic radiation and exploiting the long sample-to-detector distance; in this configuration it is possible to record the interferences patterns determined by X-rays undergoing different phase changes by passing through the sample (phase contrast). These patterns mark the borders of the details of the samples thus permitting to assess lung morphological details at high resolution, in vivo, without requiring opening of the chest wall [29]. 

The aim of the present study is to evaluate the mechanisms through which lungs microscopically deflate in experimental ARDS conditions. It does this by measuring the number and extension of airspaces at decremental volumes and in different concentric regions of interest during a stepwise lung deflation maneuver. We hypothesized that the process of lung deflation during ARDS followed the same mechanisms observed in healthy lungs during a recent synchrotron experiment [8].

## 2. Experimental Section

The care and handling of the animals followed the Directive 2010/63/EU of the European Parliament [30]. The procedures were reviewed and approved by the Internal Evaluation Committee for Animal Welfare in Research of the European Synchrotron Radiation Facility (Grenoble, France).

### 2.1. Experimental Setup

Five male New Zealand rabbits (3.7 ± 0.2 kg) underwent general anesthesia induced by an intravenous (IV) injection of sodium thiopental (25 mg/kg) and maintained by IV midazolam (0.2 mg/kg/h) and atracurium (1.0 mg/kg/h). The animals were then surgically tracheotomized using a Portex tracheal tube (no. 3, Smiths Medical, Kent, United Kingdom). A central venous catheter and an arterial line were placed into the left jugular vein and into the ipsilateral carotid artery for fluid/drugs administration and for arterial pressure monitoring. A Servo-I ventilator (Maquet, Solna, Sweden) was used to provide baseline mechanical ventilation that was delivered in pressure control mode, with a positive end-expiratory pressure (PEEP) of 3 cmH_2_O and a set pressure titrated to obtain a tidal volume (TV) of 6 mL/kg; the I:E ratio was 1:2; the FIO_2_ = 0.6. Respiratory rate was initially set at 40 bpm and then regulated to obtain a PaCO_2_ between 35 and 45 mmHg. 

A heated pneumotachograph (Hans Rudolph, Kansas City, MO, USA) was used to measure airway opening flow; respiratory volumes were computed by integrating the corresponding flow signals. Throughout the entire experiment, a dedicated data acquisition system (Powerlab, ADI Instruments, Oxfordshire, United Kingdom) sampled (4 kHz) pressure in the airways together with flow and arterial pressure, recording them on a dedicated computer.

### 2.2. Synchrotron Radiation Computed Tomography

The experiments were carried out at the Biomedical Beamline ID17 of the European Synchrotron Radiation Facility (ESRF, Grenoble, France). Image acquisition was conducted with the animal in the upright position and meeting a stationary X-ray beam while rotating on its vertical axis. The broad-spectrum synchrotron radiation was filtered by a double-crystal Si monochromator [31] to obtain a narrow energy band approximately 65 keV. Further details of the technical setup have been previously described [8,32].

### 2.3. Study Protocol

After stabilizing the animals for 30 minutes, a recruitment maneuver was performed consisting of the application of a continuous positive airway pressure (CPAP) of 20 cmH_2_O for 20 seconds. Then, the animals underwent lung injury. A two-hit ARDS model was established by performing lung lavages using warm normal saline (37 °C) until a PaO_2_/FiO_2_ ≤150 mmHg was reached. During lavages, FiO_2_ was kept at 1.0 to keep oxygen delivery compatible with animal survival. After lavage, the animals underwent injurious ventilation (the second hit) in the upright position for 120 min, setting a peak inspiratory pressure of 35 cmH_2_O, PEEP = 0, a respiratory rate of 20/min, and a FIO_2_ of 100%. At the end of injurious ventilation, a new recruitment maneuver was performed by applying a continuous positive airway pressure (CPAP) of 20 cmH_2_O for 20 seconds, and the animals were exposed to SRCT while varying their lung volume. In particular, SRCT images were acquired during end-expiratory pauses at descending PEEP levels of 12, 9, 6, 3, and 0 cmH_2_O. Between the exposures to SRCT, the animals were ventilated using a VT of 6 mL/kg, avoiding exceeding the limit of the 38 cmH_2_O plateau pressure in any of the experimental phases. In this last case, the tidal volume was diminished in order to respect this limit. The tidal volume was measured at airways opening and continuously displayed by the data acquisition system, allowing us to titrate the effective gas volume delivered to the animal. 

### 2.4. Image Recording and Analysis

During each image acquisition, 40 SRCT cranio-caudal images were acquired, each one containing an iso-gravitational slice of the lung. The imaged lung corresponded to a slice measuring 1.908 mm in the central part of the parenchyma. Image segmentation was manually performed to select only the lung parenchyma and to exclude the heart, big vessels, big airways, and chest wall. To enhance the airspaces, a sequence of top-hat transforms [33] was applied following a procedure previously described [8] and briefly summarized here. “Airspaces” were defined as the areas of the SRCT images containing gas, according to the physical density of their content. These were anatomically separated from adjacent airspaces by septal-like structures showing a tissue-like density. The number of airspaces (NAs) was calculated counting the local negative intensity peaks in the multiple top-hat final image. 

To normalize NAs for the surface, the density of the airspaces was derived by dividing the NAs by the dimension of the analyzed parenchyma, expressed as units/mm^3^ (ASnum). In particular, the analyzed parenchyma did not include the flooded or atelectatic areas that could be present on the external perimeter of the ventilated parenchyma as a consequence of lung injury. The volume subtended by each region of interest (ROI) was calculated, keeping in mind that each SRCT voxel was a cube with a side measuring 47.7 µm. The total area covered by airspaces as the total surface inside the perimeter of tissue-density boundaries was derived from the above-described image segmentation procedure. Dividing the surface covered by airspaces by their number yielded the average airspaces surface extension (ASdim). Image analysis was performed with the MatLab Image Processing Toolbox using scripts the present authors developed for the purpose.

### 2.5. Data Analysis

The analysis was carried out on the entire slice of the lung (labeled ALL) and on each of three concentric regions of interest (ROI: subpleural, mantellar, and core regions) positioned 0, 2, and 4 mm from the pleural surface, pooling the 40 consecutive slice levels sampled in each animal at each PEEP level. 

### 2.6. Histological Study

After animal sacrifice, lungs were fixed in neutral buffered formalin for 24 h, then embedded in paraffin. Hematoxylin-Eosin stained sections 4 μm thick from the lung parenchyma were retrieved with the purpose of confirming the presence of lung damage created by to the two-hit model.

### 2.7. Statistical Analysis

A linear regression analysis was performed to verify whether the single applied PEEP influenced ASdim and ASnum. Fisher’s F-test was used to compare the significant regression equations to ascertain whether the effects of PEEP were different in the different ROIs. Differences between continuous variables were assessed with the Wilcoxon signed-rank test. The set α value was 0.05 in all the statistical tests and was purposely corrected according to Bonferroni [34] when any of the tests involved multiple comparisons. 

## 3. Results

**Figure 1:**
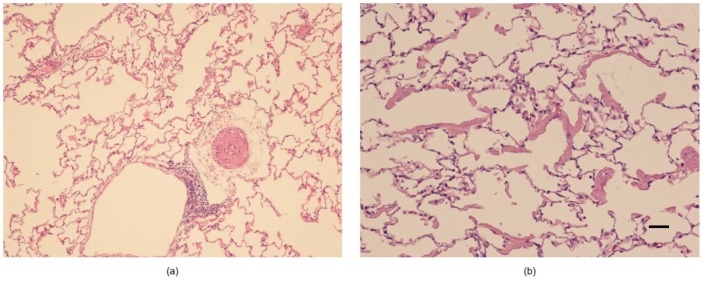
Histological samples from post-mortem biopsies. (**a**) A small artery with evident hypertrophy on the muscular layer is present in the pulmonary interstitium. (Hematoxylin-Eosin 100X original magnification); (**b**) Alveoli show an exudate with hyaline membrane appearance (Hematoxylin-Eosin 200X original magnification; bar indicates 100 μm).

All the animals survived the protocol. After the induction of lung injury, the PaO_2_/FiO_2_ ratio decrease significantly from 528 ± 52 to 115 ± 47 mmHg. The main spirometry data are reported in Table 1. The presence of ARDS alterations in the model was confirmed by the histological post-mortem analysis (Figure 1). Plateau pressure ranged from 36.3 ± 7.3 at ZEEP to 31.5 ± 6 cmH_2_O at PEEP 12.

### 3.1. Airspaces Number and Dimension in the Whole Lung Slice

The ASnum of the whole parenchyma progressively decreased while reducing PEEP (see Table 2): 188 ± 52 at PEEP 12 to 146.4 ± 96.7 at PEEP 0. Their ASdim also decreased in proportion to the applied PEEP, from 13.1 ± 2.0 to 7.6 ± 4.2 (voxels). The linear regressions between applied PEEP versus ASdim and between applied PEEP vs. ASnum were both significant. The standard deviation of both ASdim and ASnum increased with PEEP lowering (ASdim reached its maximum at PEEP 6, ASnum at PEEP 3).

### 3.2. Airspaces Number and Dimension in the Three ROIs

**Figure 2:**
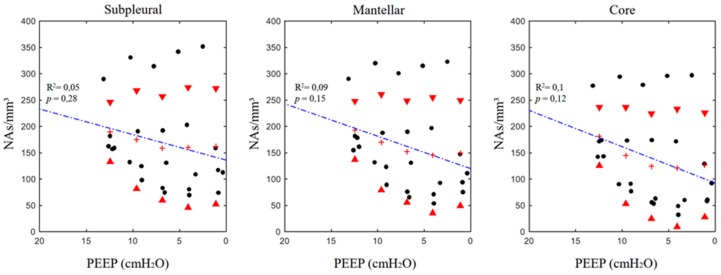
Positive end-expiratory pressure versus regional airspace numerosity (ASnum, (airpaces/mm^3^)) in the different analyzed regions of interest (ROIs). See the text for a detailed description of the computation. Red triangles mark standard deviations; crosses express the mean value at the corresponding positive end-expiratory pressure (PEEP).

**Figure 3:**
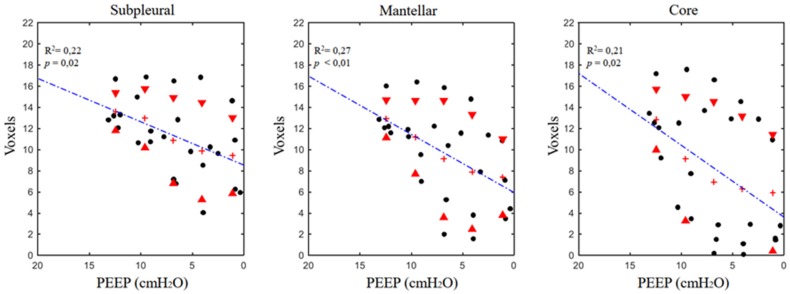
Positive end-expiratory pressure versus regional airspace dimensions expressed in voxels (1 voxel = 47.7 µm^3^) in the different analyzed ROIs. See the text for a detailed description of the computation. Red triangles mark standard deviations; crosses express the mean value at the corresponding PEEP.

The results of the regional analysis are reported in Table 2. The linear regression analysis did not show a significant decrease of ASnum in the single ROIs. A different pattern was found for ASdim, which significantly decreased in proportion to PEEP (Figure 2 and Figure 3). In the three single ROIs, the SDs of both ASdim and ASnum increased with PEEP lowering, showing that the same pattern was present in the whole slice. No significant differences in terms of ASnum could be found between the different ROIs at corresponding PEEP levels and between the regression equation expressing the relation ASdim versus PEEP.

## 4. Discussion

Based on SRCT, this study analyzed the microscopic behavior of the lung during a decreasing PEEP step maneuver in experimental ARDS conditions. For the first time, using the technological features of the synchrotron, it has been possible to assess airspace dynamics at high resolution, in vivo, during ARDS and mechanical ventilation, without requiring opening of the chest wall. Establishment of moderate ARDS was confirmed by the reduction of PaO_2_/FiO_2_ ratio and by the post-mortem histological examination. Both the peripheral and the core regions of the lung were explored in vivo during controlled mechanical ventilation. Deflation was associated with reduction in airspace dimensions both in the whole lung section and in the single ROIs. However, the regression between applied PEEP and the airspace number was significant in the whole slice but not in the single ROIs.

### 4.1. Number and Dimensional Variation in Experimental ARDS

In this model of moderate ARDS, lung deflation can be likened to a “balloon-like” behavior of the airspaces: the reduction in airspace dimensions was accompanied by a substantially constant airspace number (when regression was significant—the airspaces only increased their number in 8% of the cases) (Figure 2 and Figure 3). The other noteworthy information from the experimental data is the proportionally wide scatter of both airspace dimensions and number, particularly at the lower PEEP levels. This phenomenon is also more evident if these data are compared with the ones obtained in a similar experimental setting during healthy conditions [8].

In a two-hit ARDS model, like the one used in the present experiment, the depletion of surfactant by saline lavage generates a rise in surface tension in the alveoli [35,36]; the injurious ventilation induces a heterogeneously distributed inflammatory status that alters lung compliance and creates the basis for a further worsening of lung injury [37,38,39]. In this context, fluid bridges deriving from inflammatory edema and whose composition is modified by the depletion of surfactant (removed by lung lavage) play an important role. These fluid bridges alter the process of lung inflation/deflation during ARDS [40,41], creating local transients [42,43] and producing a heterogeneous distribution of alveolar inflation. The numerical expression of heterogeneity is the mentioned high SD of the measured ASnum and ASdim when compared with healthy lungs. Broche [44] used SRCT images coupled with an advanced model of airways dynamics including mechanical interdependency to observe the fluctuating recruitment–de-recruitment behavior of neighbor airspaces: this effect was more marked at PEEP levels lower than 6 cmH_2_O.

The mechanisms of deflation during ARDS are different from what was observed in healthy conditions [8], in which the main mechanism of deflation is the reduction of the airspace number. This implies that, in a healthy status, airspaces seem to pass between two distinct conditions, being either open or closed, while during ARDS, airspaces decrease their extension in proportion to applied PEEP. Determining how this is possible might require some reasoning on the theories behind alveolar dynamics. As early as 1979, Gil et al. [45] observed that in their microscopy preparations, the mechanism of lung deflation could be of four types: (1) sequential de-recruitment; (2) balloon-like reduction of alveolar size; (3) simultaneous change of alveolar size and shape; and (4) crumpling of the alveolar surface. However, this observation was performed in excised and fixed lungs; thus, the authors could not draw conclusions about their relative presence and/or the differences between healthy and injured lungs. Roan [46] reviewed these mechanisms more recently in the light of all the available experimental evidence; the author confirmed that the four mechanisms that Gil postulated were still valid. An important insight into how the de-recruitment mechanism of lung inflation can be more present in healthy lungs, and balloon-like behaviors more in injured lungs, derive from a seminal paper by Tsunoda in 1974 [47]. The possibility of having an “unfolding–refolding” behavior (corresponding to recruitment–de-recruitment imaging) is related to the thickness of the alveolar wall: the thinner the alveolar wall, the more “folding-bag”-like phenomena are possible. This relation is far from being simple because, when brought to high volumes, the same insufflation can thin alveolar walls. In contrast, during ARDS, thicker alveolar walls (the consequence of inflammation or intra-alveolar edema [48]) modify the resting arrangement of the single alveoli at the end expiratory lung volume, preventing their complete folding. Determinants of the different behaviors between healthy and ARDS lungs could also involve alterations of the terminal portion of the airways. Recently, the present research group [49] used SRCT to study the problem of airway closure in vivo. The study results yielded relevant findings for the present contribution: the formation of fluid bridges, the phenomenon of compliant collapse, and airway cuffing by edema are responsible for the heterogeneous distribution of alveolar opening/closing pressures present during ARDS. 

More studies are needed on this topic, and they will require an increase of time resolution imaging for SRCT scans. The different mechanisms of alveolar inflation–deflation between healthy and ARDS lungs can presumably be disclosed by imaging the few-tenths of a second of inflation starting at the end-expiratory lung volume. In fact, one speculation is that healthy airspaces, being folded and thinner than ARDS airspaces, have a very rapid initial inflation phase once the opening pressure is overcome (i.e., the “airbag model”); their transition may not be caught because of the low time resolution of the imaging. 

### 4.2. Technical Aspects and Limitations

The word “airspace” was used to indicate lung areas containing gas and that were surrounded by structures with tissue-like density. The alveoli in the rabbits had an average diameter of 110 µm [50,51]—more than double that of the SRCT pixel size used in the present experiments (47.7 µm). A sequence of top-hat functions was used because SRCT imaging resolution did not allow for the delineation of alveolar boundaries using density threshold criteria only. In this respect, top-hat functions enabled the tracing of these boundaries by simultaneously following the density and morphological criteria of local grey distribution [8]. The analyzed regions of the lung corresponded to the mid-thoracic section of the parenchyma (47.7 μm × 40 slices = 1.908 mm), and in principle, it is possible that other lung sections could exhibit different behaviors than the ones described in the present paper. For technical reasons the animals studied in the present experiment were placed vertically and the parenchyma visualized in the SRCT slices laid on the same horizontal iso-gravitational plane. This implies that the superimposed pressure [52,53] on the visualized parenchyma should be considered equally distributed in all its portions. For this reason, we could not infer any conclusion about the relation between airspace mechanics and superimposed pressure.

Addressing this issue would require properly designed experiments and a different technical arrangement capable of simultaneously imaging lung sections placed several centimeters apart on the vertical axis.

Due to the peculiarity of our setup we did not measure the end-expiratory lung volume and could not infer any conclusion about global and local strain of the lung [18] and its relation to alveolar volume variation.

We cannot report reliable measurements of esophageal pressure and consequently of transpulmonary pressure. This depends on the specificity of our experimental setup in which the mechanical perturbations by the rotating table on the gas column of the esophageal catheter is accompanied by unavoidable measurement noise. However, keeping the animals in steady state conditions, the chest wall determinants of airway pressure can be considered unaltered during the measurement phase of the experiment and do not affect the results under a qualitative point of view. 

We have studied the lung during a descending PEEP ramp sequence after an initial recruitment maneuver. The choice of passing from higher to lower intrapulmonary volumes allows to maintain the same history of volumes [54] and permits to compare directly the obtained information without needing to append a recruitment maneuver at each measurement step.

The experiments were conducted on a limited series of animals whose sample size is in line with the recommendations derived by Mead’s resource equation [55] and will require a further confirmation on a larger sample of animals. 

We have used a model of ARDS obtained by a double-hit injury, by sequentially applying lung lavage and injurious ventilation. This model has previously been found to produce inflammatory changes similar to ARDS [56]. Under the pathophysiologic point of view, the removal of surfactant creates the conditions for determining alveolar instability and damage [57]: it has been demonstrated that the effects of injurious ventilation and surfactant depletion are additive and non-reversible [58]. The local variability of injury determines a heterogeneous distribution of lung mechanical properties and paves the way for the perpetuation of damage during mechanical ventilation [3]. At a cellular level ARDS is characterized by dysregulated inflammation, accumulation of leukocytes and platelets, activation of coagulation and changes in alveolar permeability, whose molecular mechanisms have been disclosed only in part [48]. The lung injury produced by the double-hit sequence represents a model of ARDS: further studies are necessary to understand whether the findings of this study can be extended to other models of the disease or to the disease itself. Whether the results of this study can advise on the choice of the PEEP level to be applied in patients affected by ARDS [59] will require further investigation.

## 5. Conclusions

The data suggest that the macroscopic decrease in end expiratory lung volume in an animal model of moderate ARDS during a decremental PEEP trial is related to a reduction of both the dimension and number of airspaces, although the dimensional reduction is the predominant mechanism. The microscopic behavior of terminal injured airspaces is different from that of healthy lungs. Whether this structural arrangement could contribute to a further development of VILI has to be tested in properly designed experiments.

## Figures and Tables

**Table 1:** Ventilatory data. Data are expressed as mean ± SD.

Variable	PEEP 12	PEEP 9	PEEP 6	PEEP 3	ZEEP
V_T_ (mL)	33.1 ± 17.6	28.6 ± 10.9	23.4 ± 13	17.3 ± 10.7	19.9 ± 9.0
Measured PEEP (cmH_2_O)	12.4 ± 0.4	9.6 ± 0.6	6.8 ± 0.5	4.1 ± 0.7	1.1 ± 0.8
Ppeak_rs_ (cmH_2_O)	33.9 ± 5.7	32.7 ± 8.5	33.4 ± 11.1	34.1 ± 11.0	40.1 ± 7.7
Pplat_rs_ (cmH_2_O)	31.5 ± 6	29.9 ± 8.4	29.8 ± 9.8	31.7 ± 11.0	36.3 ± 7.3
Driving Pressure (cmH_2_O)	21.4 ± 5.8	23.1 ± 8.6	26.6 ± 11.0	30.0 ± 10.9	39.0 ± 7.6
C_rs_ (mL/cmH_2_O)	1.6 ± 0.8	1.4 ± 0.8	1.1 ± 0.7	0.7 ± 0.6	0.5 ± 0.3

**Table 2:** Main results.

								Parameters of Linear Regression			
		ROI	PEEP 12	PEEP 9	PEEP 6	PEEP 3	PEEP 0	m	k	R^2^	p
**Entire slice**	ASdim (voxel)	ALL	13.1 ± 2.2	11.1 ± 4.3	9 ± 5.7	8 ± 5.5	7.6 ± 4.3	0.55	6.03	0.21	<0.01 (*)
	ASnum (n/mm^3^)	ALL	188 ± 52.0	163.2 ± 86.3	145.2 ± 92.3	142.4 ± 104.9	146.4 ± 96.7	5.96	116.41	0.08	0.02 (*)
**Regional analysis**	ASdim (voxel)	SUB	13.6 ± 1.8	13 ± 2.8	10.9 ± 4.1	9.9 ± 4.6	9.4 ± 3.6	0.41	8.54	0.22	0.02 (*)
		MAN	12.9 ± 1.8	11.2 ± 3.5	9.1 ± 5.5	7.9 ± 5.4	7.4 ± 3.6	0.55	5.95	0.27	<0.01 (*)
		COR	12.9 ± 2.9	9.1 ± 5.9	6.9 ± 7.6	6.3 ± 6.9	5.9 ± 5.5	0.68	3.61	0.21	0.02 (*)
	ASnum (n/mm^3^)	SUB	189.7 ± 56.7	174.9 ± 93.3	158.7 ± 98.6	160.4 ± 114.1	162.5 ± 109.8	4.85	136.21	0.05	0.28
		MAN	193.0 ± 55.5	167.0 ± 90.9	152.3 ± 96.6	145.6 ± 109.7	149.7 ± 100.2	6.15	120.16	0.09	0.15
		COR	181.2 ± 55.5	144.7 ± 91.7	124.6 ± 99.8	121.3 ± 111.7	127.1 ± 99.1	6.89	92.86	0.10	0.12

Values expressed as mean ± SD; ROI= region of interest; PEEP = positive end-expiratory pressure; ASdim = airspaces average dimension; ASnum = airspaces density.
